# Effects of Periconception Cadmium and Mercury Co-Administration to Mice on Indices of Chronic Diseases in Male Offspring at Maturity

**DOI:** 10.1289/EHP481

**Published:** 2016-11-04

**Authors:** Cagri Camsari, Joseph K. Folger, Devin McGee, Steven J. Bursian, Hongbing Wang, Jason G. Knott, George W. Smith

**Affiliations:** 1 Laboratory of Mammalian Reproductive Biology and Genomics,; 2 Department of Animal Science,; 3 Department of Physiology,; 4 Neuroscience Program, and; 5 Developmental Epigenetics Laboratory, Michigan State University, East Lansing, Michigan, USA

## Abstract

**Background:**

Long-term exposure to the heavy metals cadmium (Cd) and mercury (Hg) is known to increase the risk of chronic diseases. However, to our knowledge, exposure to Cd and Hg beginning at the periconception period has not been studied to date.

**Objective:**

We examined the effect of Cd and Hg that were co-administered during early development on indices of chronic diseases in adult male mice.

**Methods:**

Adult female CD1 mice were subcutaneously administered a combination of cadmium chloride (CdCl_2_) and methylmercury (II) chloride (CH_3_HgCl) (0, 0.125, 0.5, or 2.0 mg/kg body weight each) 4 days before and 4 days after conception (8 days total). Indices of anxiety-like behavior, glucose homeostasis, endocrine and molecular markers of insulin resistance, and organ weights were examined in adult male offspring.

**Results:**

Increased anxiety-like behavior, impaired glucose homeostasis, and higher body weight and abdominal adipose tissue weight were observed in male offspring of treated females compared with controls. Significantly increased serum leptin and insulin concentrations and impaired insulin tolerance in the male offspring of dams treated with 2.0 mg/kg body weight of Cd and Hg suggested insulin resistance. Altered mRNA abundance for genes associated with glucose and lipid homeostasis (GLUT4, IRS1, FASN, ACACA, FATP2, CD36, and G6PC) in liver and abdominal adipose tissues as well as increased IRS1 phosphorylation in liver (Ser 307) provided further evidence of insulin resistance.

**Conclusions:**

Results suggest that the co-administration of Cd and Hg to female mice during the early development of their offspring (the periconception period) was associated with anxiety-like behavior, altered glucose metabolism, and insulin resistance in male offspring at adulthood.

**Citation:**

Camsari C, Folger JK, McGee D, Bursian SJ, Wang H, Knott JG, Smith GW. 2017. Effects of periconception cadmium and mercury co-administration to mice on indices of chronic diseases in male offspring at maturity. Environ Health Perspect 125:643–650; http://dx.doi.org/10.1289/EHP481

## Introduction

The *in utero* environment, including maternal nutrition, stress, and exposure to chemicals, can influence susceptibility of offspring to chronic diseases at adulthood (Rosenfeld 2012). The impact of environmental influences during early life on developmental programming of diseases in adulthood has been previously demonstrated in both human and animal studies (Barker et al. 2002; Ronco et al. 2011) and is linked to numerous conditions including obesity, type-2 diabetes, and hypertension (Barker 1997). During the last decade, much of the emphasis in developmental programming research has been placed on the impact of nutritional insults, endocrine disruptors, and pesticides during pregnancy (Manikkam et al. 2012; Seet et al. 2015). Less attention has been placed on *in utero* developmental programming effects of exposure to environmental contaminants such as heavy metals, cadmium (Cd) and mercury (Hg).

Previous epidemiological studies demonstrated an association between chronic Cd or Hg exposure through the diet or smoking during pregnancy and high diastolic blood pressure (Thurston et al. 2007), insulin resistance (Thiering et al. 2011), increased brain-derived neurotrophic factor concentration in cord serum (Spulber et al. 2010), and attention deficit hyperactivity disorder in children (Sagiv et al. 2012). Pronounced phenotypic effects at adulthood have also been reported in rodent studies in response to maternal and lactational Cd or Hg exposure. Gestational Cd exposure has effects on behavior (Dési et al. 1998) and on indicators of semen quality of offspring at adulthood (Petrochelli Banzato et al. 2012). Likewise, chronic maternal exposure to Hg caused altered immune and neurologic functions (Pilones et al. 2009) and behavioral effects (Onishchenko et al. 2007) in offspring.

Current data in the literature on developmental programming effects of Cd and Hg are generally derived from chronic maternal exposure to individual compounds throughout pregnancy or lactation. But, in actuality, exposure to multiple toxic chemicals may occur simultaneously from several different sources. Furthermore, due to increased public consciousness, women may change their lifestyle after becoming aware of their pregnancy to reduce potential exposures. Therefore, administration of environmental contaminants during the very early stages of pregnancy, and even before conception, is relevant to understanding the long-term effects of adverse maternal conditions on subsequent offspring health. Evidence suggests the periconception period (comprising the time immediately before and after conception) is a critical time for developmental programming (Gårdebjer et al. 2015). However, the majority of data demonstrating developmental programming during the periconception period are derived from studies that manipulated the maternal diet (Watkins et al. 2011). Studies of periconception developmental programming are of increasing importance because approximately 50% of pregnancies are unplanned in the United States, which greatly increases the chances of exposure of developing germ cells and embryo to potential unfavorable conditions during this critical time (Rayburn and Brennan 2011).

In the present study, we hypothesized that administering a combination of Cd and Hg to adult female mice during the periconception period would increase the indices of chronic disease in the offspring at adulthood. Depending on the tissue and body burden, half-lives of Cd and Hg in mice are variable (Fair et al. 1987; Feldman et al. 1978; Magos and Butler 1976). Available studies for gestational Cd or Hg exposure at varying doses during the entire pregnancy or before parturition suggest accumulation of these heavy metals mainly in maternal tissues and placenta (Barański et al. 1982; Lau et al. 1998). In contrast, only small amounts of Cd or Hg were detected in fetal organs (Hazelhoff Roelfzema et al. 1988; Lau et al. 1998). Given long half-lives of Cd and Hg, their exposure would have persisted beyond the periconception time window in dams. In addition, some direct exposure of offspring via placental transfer and lactation is also possible. Therefore, although the present study evaluated the effects of administration of Cd and Hg during the periconception period, exposure may have extended beyond this period.

## Materials and Methods

### Animals

Eight-week-old CD1 mice were obtained from Charles River Breeding Laboratories and acclimatized to local conditions for 2 weeks prior to initiation of experiments. Animals were maintained in a controlled environment with 12 hr light:dark cycle and 22 ± 1°C room temperature with food and water provided *ad libitum*. Four animals per cage were housed in standard ventilated caging system made of polysulfone where each cage was provided with autoclaved aspen-chip bedding. All animals received a commercial irradiated laboratory rodent diet (Teklad Diet 7913; Harlan Laboratories, Madison, WI). Animals were treated humanely and with regard to alleviation of suffering. All experimental procedures were approved by the Michigan State University Institutional Animal Care and Use Committee (IACUC).

## Research Design

### Experiment 1

Individually caged 10-week-old female CD1 mice were administered a combination of cadmium chloride (CdCl_2_) and methylmercury(II) chloride (CH_3_HgCl) at daily subcutaneous (sc) doses of 0.125, 0.5 or 2.0 mg of Cd and Hg each or vehicle (0.9% NaCl) for 4 days before injected females were placed with age-matched naïve males for mating (*n* = 4 litters per treatment). Subcutaneous injection was chosen as a simpler approach to better control daily amounts of Cd and Hg administered, but we acknowledge such approach is less environmentally relevant. In previous studies, administration of 0.5 mg/kg body weight of Cd (ip injection) in mice during 13–17 days of gestation (Ji et al. 2011), 2 mg/kg body weight of Cd (sc administration) in mice during 7–9 days of gestation (Paniagua-Castro et al. 2007), 2 mg/kg body weight of Hg (oral gavage) in rats during 6–9 days of gestation (Fredriksson et al. 1993), 0.5 and 2 mg/kg body weight of Hg (oral gavage) in rats during gestational day 5 until parturition (Gandhi et al. 2014) as well as 2 mg/kg body weight of Hg administration in mice at gestational day 8 (Hughes and Annau 1976) resulted in developmental programming effects in offspring. Thus dose range of 0.125–2 mg/kg body weight of Cd and Hg was selected for the current studies. Presence of a vaginal plug at embryonic day 0.5 was considered confirmation of conception, and males were separated from females. Plug-positive females were dosed as above for the 4 days following mating. Administration of Cd and Hg were performed between zeitgeber time (ZT; ZT 0 corresponds to lights on and ZT 12 corresponds to lights off in a 12 hr light:dark cycle) 4 and ZT5. Duration of adminstration of Cd plus Hg to assess developmental programming effects (4 days prior and 4 days post conception) was based on a regime used in prior studies of periconception nutritional insults (Gårdebjer et al. 2015). After completion of periconception heavy metal administration, no further Cd and Hg treatment was performed and the duration of gestation was recorded.

After delivery, birth weights were determined for all pups, and litter size was standardized to eight by randomly selecting four males and four females from each litter. Offspring were individually housed with their dams until weaning at 28 days of age. At weaning, offspring from the same litter were then group-housed together based on sex (*n* = 4 offspring/litter) and body weights were recorded throughout the duration of the experiment. Assessments of phenotypic effects of periconception Cd and Hg administration was initiated when offspring were 8 weeks of age. Endocrine and molecular indices of metabolic syndrome only were investigated in male offspring since impaired glucose homeostasis and increased abdominal adiposity were displayed together by the male, but not female offspring (described in results). Therefore, in the current study results for male offspring are emphasized.

***Behavioral tests.*** Anxiety-like behavior of male offspring was assessed by performing elevated plus maze and open field tests (Bailey et al. 2007; Rex et al. 2004). For the behavioral analyses, two males per litter were chosen randomly (*n* = 8 males per treatment). A week before the experiment, animals were individually caged and subjected to behavioral analyses at 8 weeks of age. All behavioral tests were performed between ZT4 and ZT10 (Hostetter 2013). Each animal was assigned to use only once for behavioral tests, either for elevated plus maze or open field test. The investigators were blinded to treatment status of offspring.

***Elevated plus maze test (EPM)*.** The maze consisted of a central component two opposing open arms and two opposing closed arms. Each session lasted 30 min and was videotaped. Lighting conditions and temperature of the test room were maintained constant during the entire experiment. Animals were situated individually at the center of the maze. Increased number of entries in the open arms was used for the assessment of anxiety-like behavior.

***Open field test (OFT)*.** Animals were located at the center of a mouse activity monitoring cage to determine open-field activity during 30 min sessions using the TruScan Photo Beam Activity System (Coulbourn Instruments, Whitehall, PA, USA), which is equipped with sensor rings that detect the movement of individually placed animals in every direction. The data were analyzed using TruScan 99 software (version 2.4). Decreased movement time and reduced entries to the center area as well as increased movement time in the margins and altered locomotor activity were used to assess the anxiety-like behavior.

***Glucose tolerance test (GTT).*** GTT is a well established model and was performed as described in previously published studies (Ayala et al. 2010; McIlwrath and Westlund 2015) to assess glucose homeostasis by evaluating effectiveness of clearance of glucose load administered intraperitoneally (ip). Male and female offspring at 12 weeks of age, including the offspring used previously to test behavioral analyses, were individually caged and fasted (water was provided *ad libitum*) for 6 hr in the morning before initiation of the experiment (*n* = 15 males and *n* = 14 females per treatment). GTT were performed between ZT6 and ZT10. Body weights were recorded before the experiment and 2 g/kg body weight of D-glucose (Cat. No. G7528; Sigma-Aldrich, St. Louis, MO, USA) in sterilized 0.9% NaCl was injected ip. Blood (5 μL) was obtained from the tail tip before and at 10, 20, 30, 60, 90, and 120 min after ip injection and glucose concentrations were determined with a glucometer validated for accuracy and precision prior to each experiment (True Result; Nipro Diagnostics, Fort Lauderdale, FL, USA).

***Tissue collection.*** Experiment 1 was terminated when animals reached 24 weeks of age. Tissue collections were performed between ZT3 and ZT7. Following isoflurane anesthesia, animals were euthanized by cervical dislocation. Testes, liver, kidney, and subcutaneous abdominal adipose tissues were collected and weighed (*n* = 16 males and *n* = 14 females per treatment).

### Experiment 2

A second experiment was conducted as described above to allow for assessment of additional indicators of metabolic syndrome (described below) in male offspring of treated and control dams. Individually caged 10-week-old female CD1 mice received 2.0 mg CdCl_2_ and CH_3_HgCl each sc, or vehicle (0.9% NaCl) daily for 4 days before injected females were placed with age-matched naïve males for mating (administration was performed between ZT4 and ZT5; *n* = 9 litters per treatment). As in Experiment 1, above treatments were repeated daily for 4 days following the confirmation of mating. After delivery, litter size was standardized to four males and four females from each litter and offspring were housed together with their dams until weaning at 28 days of age. At weaning, offspring from the same litter were then group-housed together based on sex (4 males/litter, 36 males/treatment). A single 2.0 mg/kg dose was selected as it elicited the most striking phenotypic changes across all endpoints examined in Experiment 1.

***Insulin tolerance test (ITT).*** ITT was performed as described in previously published studies (Head et al. 2012) to assess insulin sensitivity via measuring blood glucose concentrations upon ip administration of insulin. Thirteen-week-old male offspring of control and treated females were individually caged and fasted (water was provided *ad libitum*) for 6 hr in the morning before starting the experiment (*n* = 34 for controls and *n* = 35 for treated offspring; two offspring in control and one offspring in treatment group were excluded from the test due to signs of excessive stress at initiation of experiment). ITT were performed between ZT5 and ZT9. Then, 0.75 IU/kg body weight of human recombinant insulin (Novolin-R, Novo Nordisk, Plainsboro, NJ, USA) was administered ip. Blood (5 μL) was obtained from the tail tip before and at 10, 20, 30, 60, 90, and 120 min after ip injection and glucose concentrations were determined with a glucometer (True Results) as described for glucose tolerance test.

### Blood and Tissue Collection

Experiment 2 was terminated at two different time points when male offspring were 13 and 25 weeks of age. Animals were randomly assigned to termination groups either at 13 or 25 weeks of age. However, blood and tissue samples obtained at 13 weeks of age were saved for further investigation. In the present studies, blood and tissue samples were only obtained from offspring at 25 weeks of age (*n* = 19 for controls and *n* = 17 for treated offspring). Blood and abdominal adipose tissue collections were performed between ZT3 and ZT7. Blood was collected at room temperature from the retro-orbital sinus under isoflurane anesthesia. Then, blood samples were centrifuged at 5,000 *g* for 15 min and serum was stored at –80°C. After collecting blood, animals were euthanized by cervical dislocation and abdominal adipose tissue was collected and weighed.

### Hormone Assays

Serum samples were shipped to the Endocrine Technologies Support Core at the Oregon National Primate Research Center (Beaverton, OR, USA) for determination of leptin and insulin concentrations using validated assays (Rull et al. 2014; Wang et al. 2013). Blood samples were obtained from offspring when Experiment 2 was terminated at 25 weeks of age (*n* = 19 for controls and *n* = 17 for treated offspring). Samples were analyzed by enzyme-linked immunosorbent assay (ELISA; Mouse Leptin, Millipore, Billerica, MA, USA; Mouse Insulin, Mercodia, Winston-Salem, NC, USA). Intra- and inter-assay coefficients of variation were 6.9% and 7.86%, respectively, for leptin and 1.67% and 2.25%, respectively, for insulin.

### RNA Isolation and Real Time PCR Analysis

In adipose tissue, expression of genes involved in glucose uptake (GLUT4), transmission of insulin signaling (IRS1), and fatty acid synthesis (ACACA and FASN) was analyzed. Reduced abundance of mRNA for these genes is correlated with insulin resistance and increased adiposity (Dharuri et al. 2014; García-Fuentes et al. 2015; Mesnier et al. 2015; Wang et al. 2012). Likewise, in the liver, transcript abundance for genes required for fatty acid uptake (FATP2 and CD36) and glucose homeostasis (G6PC) was analyzed in male offspring of control and treated females. Aberrant mRNA expression for these genes is positively correlated with insulin resistance and impaired glucose homeostasis (Ge et al. 2010; Konstantynowicz-Nowicka et al. 2015; Lee et al. 2015) (*n* = 11 for controls and *n* = 17 for treated offspring for both adipose and liver tissues; at the termination of the experiment, tissue samples were randomly chosen for molecular studies and all available samples were used).

Total RNA was isolated from adipose and liver tissue of male offspring using an RNeasy® Mini kit (Qiagen, Valencia, CA, USA) based on the manufacturer’s instructions. Following DNase treatment for removal of genomic DNA contamination, RNA samples were reverse transcribed using iScript™ cDNA synthesis kit (BioRad, Hercules, CA, USA) based on instructions. After completion of cDNA synthesis, samples were diluted to a final volume of 100 μL with nuclease-free water.

Real-time quantitative polymerase chain reaction (RT-PCR) was used for the quantification of gene transcripts (CFX96TM RT-PCR System BioRad). PerlPrimer® Software (http://perlprimer.sourceforge.net) v1.1.21 (Marshall 2004) was used to design all the PCR primers used in the present studies (see Table S1). Transcript abundance for genes of interest was normalized using the ΔΔCT method. This method requires an internal control gene that is expressed similarly between control and experimental treatment. In the present studies, hypoxanthine phosphoribosyltransferase (HPRT) was chosen as the reference gene both for adipose and liver tissue. Initial tests showed that transcript abundance for HPRT in adipose and liver tissue was similar between control and treatment group offspring (*p* > 0.05; *n* = 11 for controls and *n* = 17 for treated offspring).

### Western Blot Analyses

In previously published studies, phosphorylation of insulin receptor substrate 1 (IRS1) at serine 307 in liver has been shown to be associated with insulin resistance (Koketsu et al. 2008). Therefore, in the present studies IRS1 phosphorylation at serine 307 was investigated in liver tissue for the subset of randomly selected male offspring (*n* = 11 for controls and *n* = 17 for treated offspring; at the termination of the experiment, tissue samples were randomly chosen for molecular studies and all available samples were used). Samples were homogenized in RIPA buffer (150 mM NaCl, 1% IGEPAL®, 0.5% sodium deoxycholate, 0.1% SDS, and 50 mM Tris, pH 8.0) containing 1× protease and phosphate inhibitor cocktail (Roche Applied Science) and protein concentration was determined by Bradford protein assay (BioRad). Samples (10 μg protein) were separated via SDS-polyacrylamide gel electrophoresis (Bio-Rad) and transferred to polyvinylidene difluoride (PVDF) membranes (Millipore, Bedford, MA, USA). Following blocking with 5% BSA blocking buffer prepared in Tris buffered saline with Tween 20® (TBST; 137 mM sodium chloride, 20 mM Tris, 0.5% Tween 20), membranes were incubated overnight with respective primary antibodies against phosphorylated insulin receptor substrate 1 (p-IRS1; phosphorylated at Ser 307; rabbit polyclonal, Santa Cruz Biotech; sc-101709), total-IRS1 (T-IRS1; C-20; rabbit polyclonal, Santa Cruz Biotech; sc-559) and total actin (clone C4; mouse monoclonal; Millipore; MAB1501), respectively. Following washing with TBST, membranes were incubated with HRP-conjugated anti-rabbit IgG for p-IRS1 and T-IRS1 (Anti-rabbit IgG; Cell Signaling Technology; 7074) and anti-mouse IgG for total actin (Goat anti-mouse IgG; Thermo Scientific; 31430). Super Signal West Dura Chemiluminescent Substrate (Thermo Scientific, Waltham, MA, USA) was used to visualize protein bands using a myECL imager (Thermo Scientific). Density of the protein bands were determined by using ImageJ software (Schneider et al. 2012). Protein abundance was determined by normalizing the data relative to abundance of total actin.

### Statistical Analyses

In the present studies, due to potential differences in susceptibility of adverse *in utero* conditions between each sibling within a litter, individual offspring were used as an experimental unit (Chen et al. 2015; Ngo et al. 2014). Effects on birth weights, litter size, organ weights, area under the curve, hormone concentrations, differences in mRNA expression, and open arm entries in the EPM were analyzed using a one-way ANOVA with Proc GLM in SAS (version 9.2; SAS Institute, Cary, NC, USA). When the ANOVA test was significant, differences between least square means for treated animals versus controls were analyzed with Dunnett’s multiple comparison test. Differences in body weights, glucose tolerance, insulin resistance, locomotor activity, and time spent at the center area and the margin walls in the OFT were tested using repeated measures ANOVA. Statistical significance was based on *p* < 0.05. Data are expressed as mean ± standard error of the mean (SEM).

## Results

### Gestation Length, Litter Size and Birth Weights (Experiment 1)

There were no significant differences from controls in gestational length, litter size or mean birth weights of offspring in any treatment group (*p* > 0.05; see Table S2).

### Behavioral Tests

**Figure 1:**
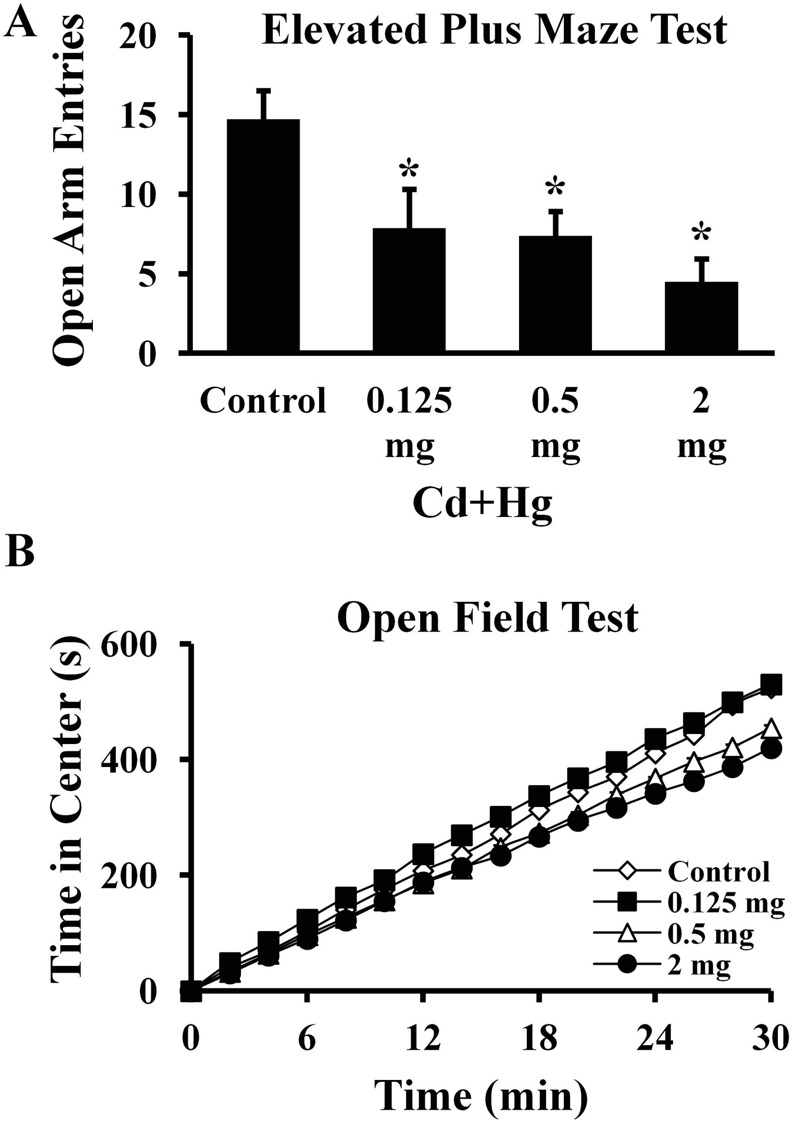
Anxiety-like behavior of 8-week-old male offspring. Open arm entries and cumulative amount of time spent in the center area were tested by EPM and OFT, respectively (*p < 0.05 compared with controls; *n *= 8 animals per treatment). (*A*) Open arm entries. The *x*-axis represents each treatment group. Data are presented as mean ± SEM. (*B*) Cumulative amount of time spent in the center area. The *x*-axis represents total experimental duration of OFT. Significant differences over time between treatment and control offspring were tested.

Offspring from all treatment groups had reduced open arm entries in EPM compared with controls (*p* < 0.05; Figure 1A). Male offspring from females treated with 0.5 and 2.0 mg/kg body weight of Cd and Hg spent more time in the margin area (*p* < 0.05; see Figure S1A) and less time in the center area (*p* < 0.05; Figure 1B) of the OFT compared with controls. Likewise, offspring from females administered 2.0 mg/kg body weight of Cd and Hg exhibited lower numbers of entries to the center area (*p* < 0.05; see Figure S1B; *n* = 8 males per treatment). However, locomotor activities of treated males (assessed by total distance travelled in the OFT) were similar compared with controls (*p* > 0.05; see Figure S2).

### Glucose Tolerance Tests (Experiment 1)

**Figure 2:**
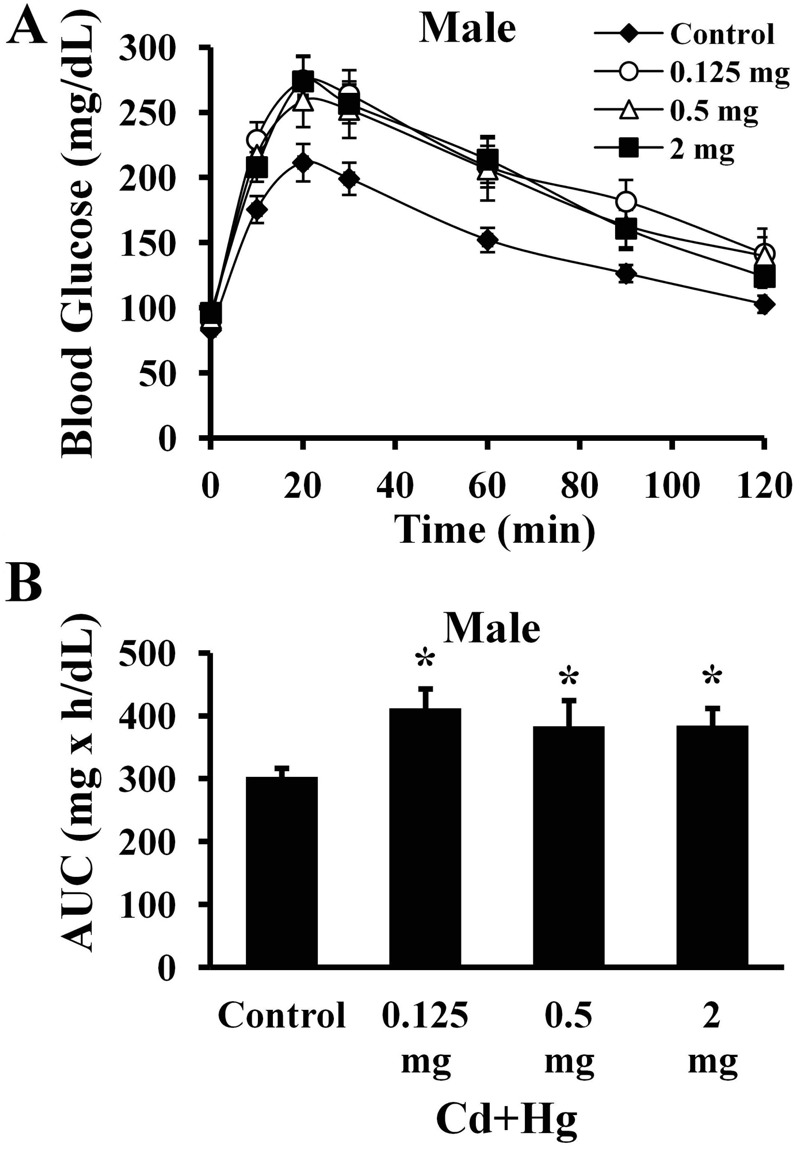
GTT and AUC values for 12-week-old male offspring. (*A*) Glucose tolerance of male offspring (*n *= 15 per treatment). The *x*-axis represents experimental duration in minutes and the *y*-axis represents blood glucose concentration in mg/dL. Significant differences over time between treatment and control offspring were tested (*p *< 0.05 compared with controls). (*B*) AUC values (*p < 0.05 compared with controls). The *x*-axis represents each treatment group. The *y*-axis represents AUC values for plasma glucose in mg × hr/dL of blood. Note: Data are presented as mean ± SEM.

Peak glucose levels were reached 20 min following glucose administration and remained higher until 120 min post-glucose challenge in treatment group offspring relative to controls (*p* < 0.05; Figure 2A). Area under the curve (AUC) was also calculated, which depicts cumulative concentrations of glucose in the blood relative to baseline fasting concentrations. The AUCs were also greater for male offspring from all three treatment groups compared with controls (*p* < 0.05; Figure 2B; *n* = 15 males per treatment).

Female offspring of female mice injected with Cd plus Hg at all three doses tested had elevated blood glucose levels compared with controls, with peak levels 20 min after administering an ip bolus of glucose (*p* < 0.05; see Figure S3A). Likewise, AUC values were significantly higher for female offspring from all treatment groups compared with controls (*p* < 0.05; see Figure S3B; *n* = 14 females per treatment).

### Body and Adipose Weights

**Figure 3:**
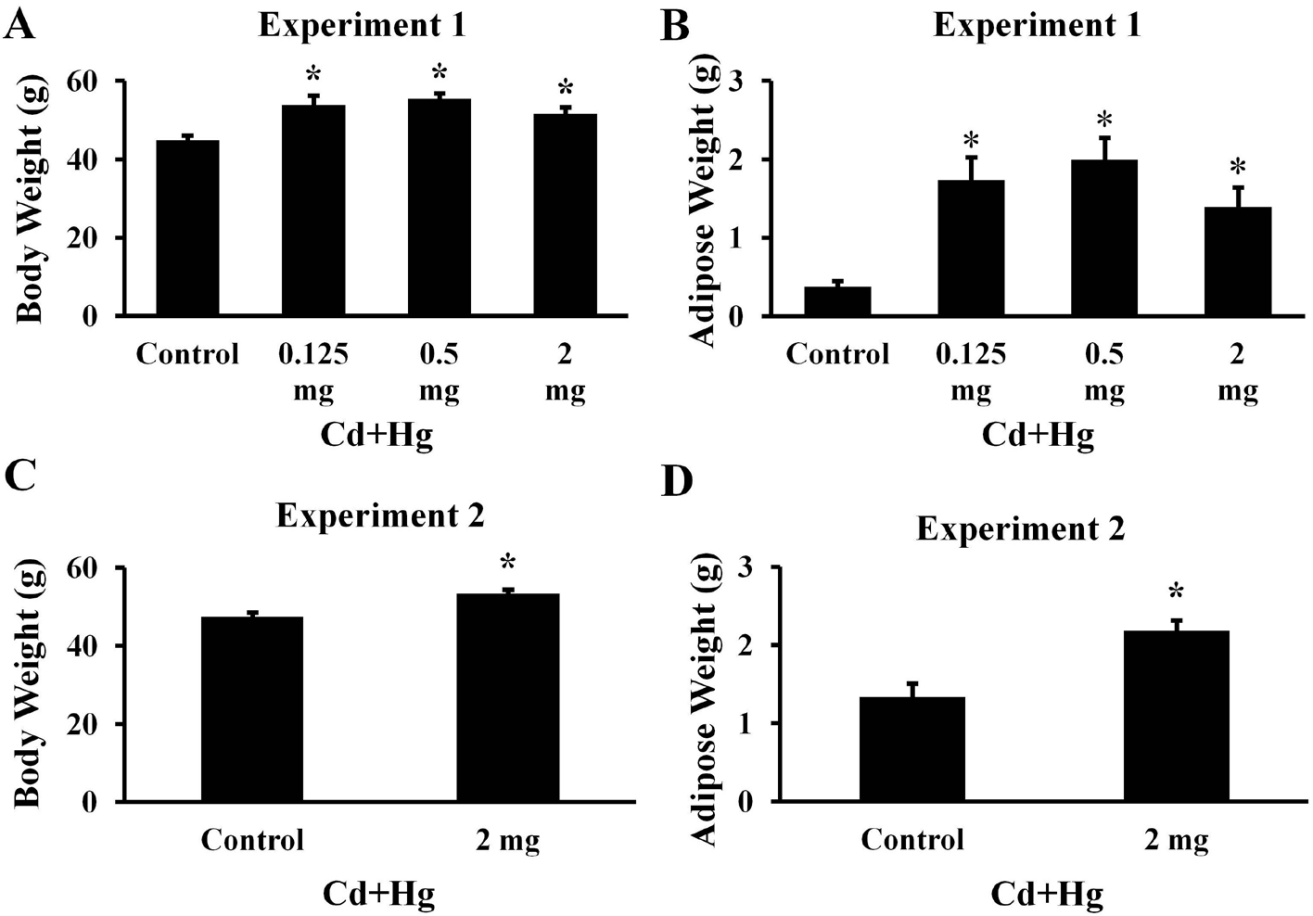
Body and adipose weights of male offspring from (*A*, *B*) Experiment 1 at 24 weeks of age and from (*C*, *D*) Experiment 2 at 25 weeks of age (*p < 0.05 compared with controls; *n *= 16 per treatment in Experiment 1; *n *= 19 for controls and *n *= 17 for treated offspring in Experiment 2). Left panels (*A, C*) show offspring body weights. Right panels (*B, D*) shows offspring abdominal adipose weights. The *x*-axis represents each treatment group. Note: Data are presented as mean ± SEM.

Body weights were similar at weaning. For offspring in Experiment 1, there were no significant differences in body weights until 15 weeks of age, but weights were significantly higher in all treatment groups compared with controls after 15 weeks of age until the experiment terminated at 24 weeks of age (*p* < 0.05; Figure 3A; see also Table S4; *n* = 16 males per treatment). At 24 weeks of age, abdominal adipose weights were higher in all treatment groups compared with controls (*p* < 0.05; Figure 3B). Organ weights were only measured in Experiment 1, and there were no significant differences in liver, testes, or kidney weights (*p* > 0.05; see Table S3). Treated mice in Experiment 2 also had significantly higher mean body weight and adipose tissue weight than controls measured at 25 weeks of age (*p* < 0.05; Figure 3C,D see Table S5; *n* = 19 for controls and *n* = 17 for treated offspring).

*In utero* treatment with Cd plus Hg did not affect body and adipose weights of female offspring at 24 weeks of age compared with controls (*p* > 0.05; see Figure S4A,B *n* = 14 females per treatment).

### Markers of Insulin Resistance (Experiment 2)

**Figure 4:**
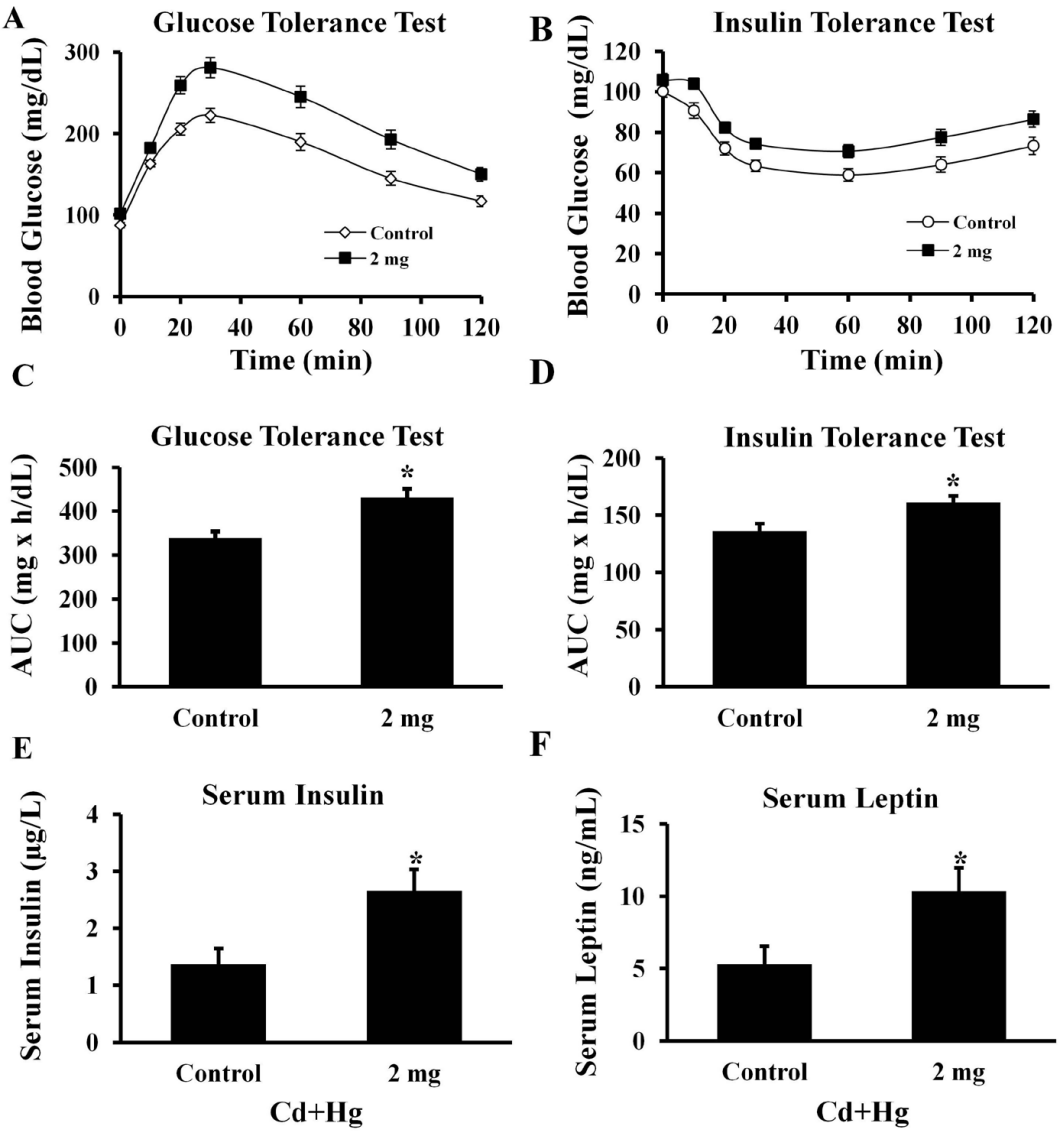
Indices of metabolic syndrome for male offspring in Experiment 2. (*A*) GTT results at 12 weeks of age for 34 male controls and 35 male offspring of dams treated with 2.0 mg/kg Cd and Hg. (*B*) ITT results at 13 weeks of age for 34 male controls and 35 male offspring of dams treated with 2.0 mg/kg Cd and Hg. Significant differences over time between treatment and control offspring were tested in (*A*) and (*B*) (*p *< 0.05 compared with controls). (*C*) GTT results expressed as the AUC for each group. (*D*) ITT results expressed as the AUC for each group. (*E*) Serum insulin concentrations in μg/L at 25 weeks of age for 19 male controls and 17 male offspring of dams treated with 2.0 mg/kg Cd and Hg. (*F*) Serum leptin concentrations in ng/mL at 25 weeks of age for 19 male controls and 17 male offspring of dams treated with 2.0 mg/kg Cd and Hg. Note: All values are mean ± SEM (*p < 0.05 compared with controls); data are presented as mean ± SEM.

Male offspring obtained during Experiment 2 were subjected to GTT and ITT starting at 12 weeks of age. As reported in Experiment 1, treatment group males had reduced glucose tolerance relative to controls (*p* < 0.05; Figure 4A,C). Following insulin administration (0.75 IU/kg body weight), higher concentrations of blood glucose were observed in male offspring of treated females (*n* = 35 offspring) compared with controls (*n* = 34 offspring) at all time points tested (*p* < 0.05; Figure 4B). Likewise, AUC was greater for male offspring of treated females compared with controls demonstrating that total amounts of glucose in circulation were significantly higher (*p* < 0.05; Figure 4D). Following a 6-hr fast, serum concentrations of insulin (*p* < 0.05; Figure 4E) and leptin (*p* < 0.05; Figure 4F) were significantly elevated in male offspring derived from treated females compared with controls (*n* = 19 for controls and *n* = 17 for treated offspring).

### mRNA Abundance

**Figure 5:**
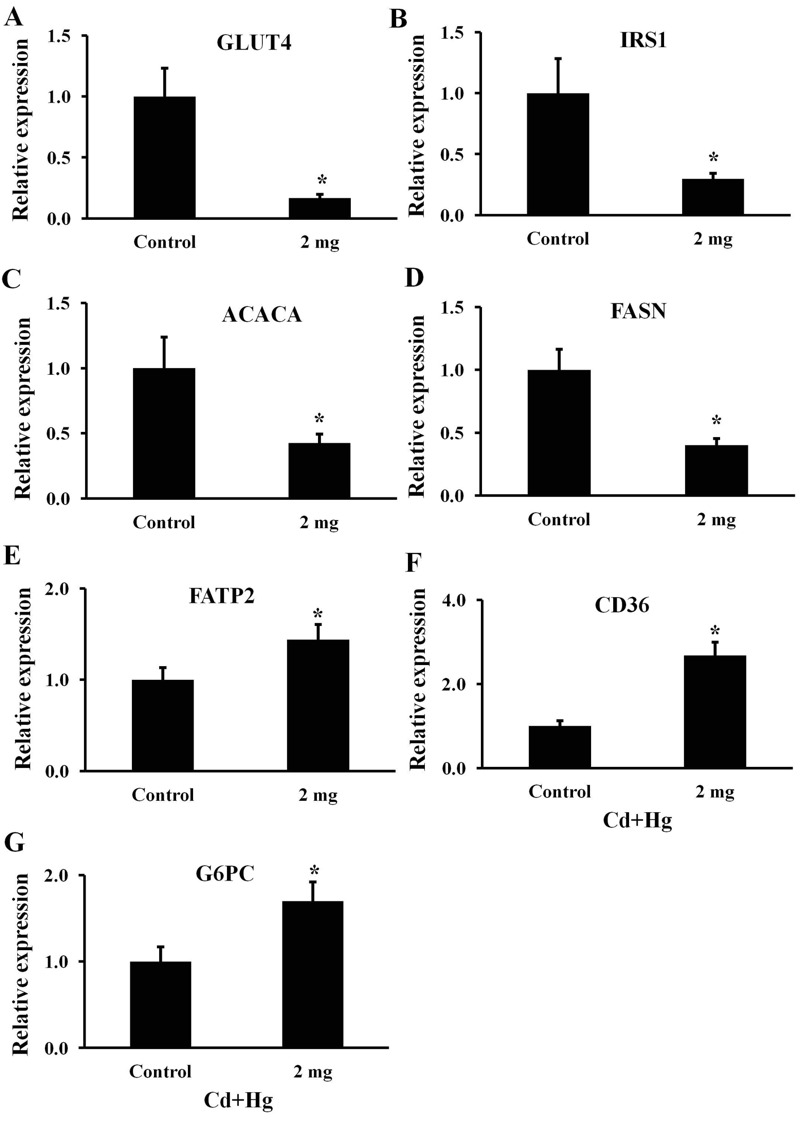
Effect of periconception Cd and Hg administration on mRNA expression at 25 weeks of age in male offspring of control dams (*n* = 11) and the male offspring of dams treated with 2.0 mg/kg Cd and Hg (*n* = 17) for (*A*) GLUT4, (*B*) IRS1, (*C*) ACACA, and (*D*) FASN in abdominal adipose tissue and for (*E*) FATP2, (*F*) CD36, and (*G*) G6PC in liver tissue. Note: Data are presented as mean ± SEM. *p < 0.05 compared with controls. ACACA, acetyl-CoA carboxylase alpha; CD36, molecule thrombospondin receptor; FASN, fatty acid synthase; FATP2, fatty acid transporter, solute carrier family 27, member 2; G6PC, glucose-6-phosphatase; GLUT4, glucose transporter type 4; IRS1, insulin receptor substrate 1.

Real time PCR analysis showed a significant reduction in mRNA abundance for GLUT4, IRS1, ACACA and FASN in adipose tissue of male offspring of treated females compared with controls (*p* < 0.05; Figure 5A–D; *n* = 11 for controls and *n* = 17 for treated offspring).

There was increased mRNA abundance for FATP2, CD36, and G6PC in male offspring of treated females compared with controls (*p* < 0.05; Figure 5E–G; *n* = 11 for controls and *n* = 17 for treated offspring). Phosphorylation of insulin receptor substrate 1 (IRS1) at serine 307 in liver tissue, which has been associated with insulin resistance (Koketsu et al. 2008), was significantly higher in male offspring of the 2.0 mg/kg Cd and Hg treatment group (*p* < 0.05; mean ratio of p-IRS1 to total IRS1 0.66 ± 0.04, *n* = 17 mice) than in vehicle controls (0.30 ± 0.04, *n* = 11 mice).

## Discussion

In our studies, we investigated the potential effect (using an animal model) of maternal exposure to heavy metals (administered during the periconception period) on offspring susceptibility to adult onset chronic diseases. In the present study, the adult male offspring of mice that received Cd and Hg during the periconception period had evidence of increased anxiety-like behavior, higher body weight, higher abdominal adipose weight, and impaired glucose homeostasis. To our knowledge, this is the first study demonstrating phenotypic changes in body and adipose weight and insulin resistance in mice administered Cd and Hg during early development. In previous rodent studies, ip injection of 0.5 mg/kg body weight of Cd (13–17 days of gestation) and sc administration of 2 mg/kg body weight of Cd (7–9 days of gestation) resulted in developmental programming effects in offspring (Ji et al. 2011; Paniagua-Castro et al. 2007). Likewise, similar developmental programming effects in offspring were also observed after *in utero* administration of 0.5 and 2 mg/kg body weight of Hg (gestational day 5 until parturition or 6–9 days of gestation) via oral gavage or injecting 2 mg/kg body weight of Hg at gestational day 8 (Fredriksson et al. 1993; Gandhi et al. 2014; Hughes and Annau 1976). These studies provided the basis for the selection of dose range as 0.125–2 mg/kg body weight in the present studies. Current studies in the literature with exposure to Cd or Hg individually at varying doses (0.5–10.0 mg/kg body weight) during the entire gestation period or after embryo implantation often report decreased birth weights (Beyrouty and Chan 2006; Hughes and Annau 1976; Jacquillet et al. 2007; Ji et al. 2011). However, body weight changes or increased adiposity at adulthood were not reported in these studies (Beyrouty and Chan 2006; Hughes and Annau 1976; Jacquillet et al. 2007; Ji et al. 2011). To our knowledge, impaired glucose homeostasis and insulin resistance also has not been examined previously in response to prenatal Cd or Hg administration.

Results obtained in the EPM and OFT demonstrate anxiety-like behavioral effects without an impact on locomotor activity. Similar locomotor activity observed between male offspring of treated and control dams suggest increased anxiety-like behavior was not linked to lethargy or reduced activity of offspring. In previous studies, increased anxiety-like behavior was observed with administration of Hg either at a very high dose, such as 8.0 mg/kg body weight on gestational day 8 (Maia et al. 2009), or administration later in gestation corresponding to the stage of organ and nervous system development where Cd and Hg might have direct impacts on offspring behavior. For instance, offspring of pregnant mice fed a diet corresponding to Hg at a daily dose of 0.01 mg/kg body weight from gestational days 8 to 18 spent less time in the open field area suggesting increased anxiety-like behavior (Montgomery et al. 2008). In contrast, reduced anxiety-like behavior reflected by increased open arm entries in the EPM was reported in offspring from mothers exposed to 0.6 mg/kg body weight of Cd from gestational days 7 to 15 (Minetti and Reale 2006). However, in the present studies Cd and Hg administration started 4 days before conception and ceased before the normal day of embryo implantation. While it is possible that the apparent effect on anxiety-like behavior was the consequence of developmental programming rather than a direct impact of Cd and Hg on the organ systems of the developing fetus, it is not possible to rule out direct exposure through placental transfer (Hazelhoff Roelfzema et al. 1988; Lau et al. 1998) and possibly exposure of nursing pups after birth (Bekheet 2011; Petersson Grawé et al. 2004), given the long half-lives of Cd and Hg (Fair et al. 1987; Feldman et al. 1978; Magos and Butler 1976).

Metabolic syndrome (MS) is a cluster of conditions, including altered glucose homeostasis and abdominal obesity, that increase the risk of type-2 diabetes and cardiovascular disease (Huang 2009). Our data suggest that MS is triggered in male offspring of treated females. Impaired glucose tolerance of offspring could be caused by reduced sensitivity to insulin that maintains normal blood glucose homeostasis. To examine the indices of MS in male offspring of females exposed to Cd plus Hg at periconception, a series of endocrine and molecular indices were measured. Since all doses of Cd plus Hg caused impaired glucose tolerance and increased adiposity in male offspring, female mice were treated with only the 2.0 mg/kg body weight dose of Cd plus Hg during the periconception period (Experiment 2). Insulin tolerance studies performed at 13 weeks of age indicated altered glucose homeostasis is caused by reduced sensitivity to insulin. Similar to the previous experiment, male offspring also exhibited increased body weight and abdominal adipose accumulation. Results suggest that exposure to Cd plus Hg, or possibly Cd alone or Hg alone, beginning 4 days before conception and 4 days following mating reduced the cellular response of metabolic tissues to insulin. This might result in reduced glucose uptake and utilization by metabolic tissues that could cause build up of excess glucose in the systemic circulation over time as seen in the development of type 2 diabetes (Alsahli and Gerich 2012). At this stage of insulin resistance, reduction of insulin sensitivity could still be adequate to keep fasting blood glucose in normal range, but when challenged with a meal or a glucose upload, postprandial glucose tolerance becomes abnormal (Alsahli and Gerich 2012). Therefore, as a compensatory mechanism, pancreatic β-cells secrete more insulin into the circulation (Shanik et al. 2008). Consequently, increased serum insulin concentrations suggestive of insulin resistance were observed in the current studies. Alterations in mRNA abundance for GLUT4, IRS1, ACACA, FASN, FATP2, CD36, and G6PC observed in adipose and liver tissues, coupled with elevated insulin and leptin in male offspring, provide further evidence for developmental programming of MS in this model, though as noted previously, exposure may have continued after birth due to placental or lactational transfer.

Results of the described studies show clear differences from controls in multiple indices of chronic disease in the adult offspring of dams that were subcutaneously administrated Cd and Hg daily 4 days before and 4 days after conception. An important question that remains to be answered is the potential intracellular mechanism(s) of action whereby heavy metals elicit developmental programming effects. Offspring phenotypic changes in response to maternal nutrient restriction have been linked to the perturbation of cell cycle in whole embryo leading to diminished integrity of DNA at the time of nutritional insult (Swali et al. 2011). Alternatively, it is possible that observed phenotypic effects are mediated, at least in part, by the exposure of oocytes/embryos to heavy metals elicited with the periconception administration regime utilized. However, observed offspring phenotype could also be attributed to Cd and Hg that persist in the maternal circulation and target tissues during the later stages of gestation because of their long half-lives. Because of these toxicokinetic properties, future experiments involving embryo transfer, where transfer of embryos from females administered Cd plus Hg to naïve pseudo-pregnant recipients, and vice versa, will be required to conclusively prove that developmental programming effects observed in the current studies are attributed, at least in part, to exposure during the periconception period. Moreover, future experiments should also compare outcomes in mice treated with both Cd and Hg with outcomes in mice treated with Cd alone and Hg alone.

## Conclusion

The present studies provide novel findings regarding anxiety-like behavior and indices of chronic disease, including impaired glucose homeostasis, insulin resistance, and increased body weight, in adult male offspring of CD-1 mice treated with Cd and Hg for 4 days before and after conception. Results support further investigation of the effects of periconception exposure to heavy metals and other toxicants, on incidence of chronic disease and mechanisms involved.

## Supplemental Material

(500 KB) PDF
