# Supplementary material for: Effects of Periconception Cadmium and Mercury Co-Administration to Mice on Indices of Chronic Diseases in Male Offspring at Maturity
Source: Environ Health Perspect. 2016 Nov 4;125(4):643–50. doi: 10.1289/EHP481 (PMC5381999; doi:10.1289/EHP481)
Supplement: (500 KB) PDF [file EHP481.s001.acco.pdf]

Environ Health Perspect
DOI: 10.1289/EHP481

Note to readers with disabilities: *EHP* strives to ensure that all journal content is accessible to all readers. However, some figures and Supplemental Material published in *EHP* articles may not conform to 508 standards due to the complexity of the information being presented. If you need assistance accessing journal content, please contact ehponline@niehs.nih.gov. Our staff will work with you to assess and meet your accessibility needs within 3 working days.

Supplemental Material

Effects of Periconception Cadmium and Mercury Co-Administration to Mice on Indices of Chronic Disease in Male Offspring at Maturity

Cagri Camsari, Joseph K. Folger, Devin McGee, Steven J. Bursian, Hongbing Wang, Jason G. Knott, and George W. Smith

Table of Contents

Table S1: The list of primers used

Table S2: Birth weights and litter size of offspring of control and periconception Cd plus Hg-treated female mice and impact on gestational length.

Table S3: Organ weights of 24-week-old male offspring of control and periconception Cd plus Hg-treated female mice.

Table S4: Body weights of male offspring of control and periconception Cd plus Hg-treated female mice in Experiment 1.

Table S5: Body weights of male offspring of control and periconception Cd plus Hg-treated female mice in Experiment 2.

Figure S1: Anxiety-like behavior of eight-week-old male offspring of control and periconception Cd plus Hg-treated female mice.

Figure S2: Locomotor activity of male offspring at eight weeks of age.

Figure S3: Glucose tolerance test and area under the curve values for 12-week-old female offspring.

Figure S4: Body and adipose weights of female offspring at 24 weeks of age.

Primer	Forward Sequence (5'-3')	Reverse Sequence (5'-3')	Product Size (bp)
**GLUT4**	CTACTCAGGGCTAACATCAGG	CAACCAGAATGCCAATGACGA	118
**IRS1**	CACCATCTCAACAACCCTCC	GTTTCCCACCCACCATACTG	102
**ACACA**	GAAGCCAAGATAATCCAGCA	CATACATATCTTTCATCCCACCAG	168
**FASN**	TGGGTGTGAGTGGTTCAGAG	CAATGCTTGGTCCTTTGAAGTC	140
**CD36**	TGGCTAAATGAGACTGGGAC	GCAACAAACATCACCACTCC	130
**FATP**	GAAGTGAATGTGTATGGCGTG	CTCAATGGTATCTTGTATCCTCAG	173
**G6PC**	GCCTTCTATGTCCTCTTTCCC	AACAGAATCCACTTGAAGACGAG	115

Table S1: List of primers used and listed in forward and reverse direction (5'-3'). Altered mRNA abundance of specific genes was used to assess the impact of periconception Cd and Hg administration on glucose and lipid metabolism in liver and abdominal adipose tissues.

		Treatment			
		**Control**	**0.125 mg**	**0.5 mg**	**2 mg**
**Birth Weight (g)**		1.58 ± 0.03 (n=55)	1.61 ± 0.02 (n=51)	1.57 ± 0.02 (n=55)	1.63 ± 0.02 (n=53)
**Litter Size**	**Litter 1**	18	14	14	14
	**Litter 2**	12	15	14	12
	**Litter 3**	14	12	13	16
	**Litter 4**	11	10	14	11
**Gestational Length (Days)**	**Litter 1**	18	18	18	19
	**Litter 2**	18	18	18	19
	**Litter 3**	19	19	19	19
	**Litter 4**	19	19	18	19

Table S2: Birth weights and litter size of male and female offspring of control and periconception Cd plus Hg-treated female mice and gestation length. There were no significant differences in any of the parameters tested between individual treatment groups and controls. ($P>0.05$). Data are presented as mean ± SEM.

Treatment	Organ Weights at 24 Weeks of Age		
	**Liver**	**Testes**	**Kidney**
**Control**	2605.41 ± 112.40	271.52 ± 9.33	902.36 ± 37.39
**0.125 mg**	2723.38 ± 196.19	251.62 ± 6.45	863.47 ± 35.20
**0.5 mg**	2828.71 ± 110.28	303.91 ± 11.63	939.79 ± 40.38
**2 mg**	2469.88 ± 90.53	253.46 ± 12.11	958.48 ± 22.73

Table S3: Organ weights of 24-week-old male offspring of control and periconception Cd plus Hg-treated female mice. There were no significant differences in any of the parameters tested between individual treatment groups and controls. ($P>0.05$; $n=16$ offspring per treatment). Data are presented as mean ± SEM.

Treatment	Week12	Week 15	Week 18	Week20	Week22	Week 24
**Control**	45.55	46.55	45.66	45.51	44.63	44.89
**0.125 mg**	49.46	50.21	51.59*	52.27*	52.16*	54.86*
**0.5 mg**	51.19	51.11	51.53*	51.51*	53.66*	57.30*
**2 mg**	49.43	49.89	50.50*	49.61*	50.17*	51.66*

Table S4: Body weights of male offspring of control and periconception Cd plus Hg-treated female mice in Experiment 1. There were no significant differences in body weights until 15 weeks of age, but body weights were significantly higher in all treatment groups compared to controls after 15 weeks of age until the experiment terminated at 24 weeks of age (* $P<0.05$ compared to controls; n=16 offspring per treatment).

Treatment	Week13	Week 16	Week 17	Week 22	Week 25
**Control**	43.09	45.59	46.56	51.16	51.01
**2 mg**	42.79	46.59	49.25	54.07*	57.44*

Table S5: Body weights of male offspring of control and periconception Cd plus Hg-treated female mice in Experiment 2. Body weights were significantly higher in the treatment group male offspring compared to controls at 22 and 25 weeks of age (* $P<0.05$ compared to controls; n=19 for controls and n=17 for treated offspring).

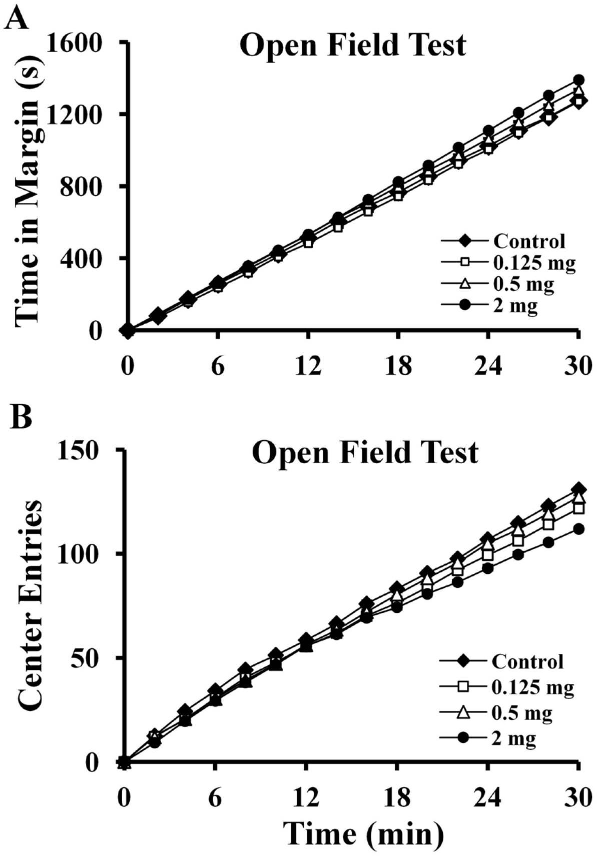

Figure S1: Anxiety-like behavior of eight-week-old male offspring of control and periconception Cd plus Hg-treated female mice. Cumulative amount of time spent in the margin area and entries to the center area were tested by open field tests. Significant differences over time between treatment versus control offspring were tested. ($P<0.05$ compared to controls; n=8 animals per treatment). (A) Cumulative amount of time spent in the margin area for male offspring. Offspring of 0.5 and 2 mg treatment groups spent significantly more time in the margin area compared to controls (B) Cumulative amount of entries to the center area for male offspring. Offspring of 2 mg treatment group displayed significantly reduced number of entries to the center area compared to controls. X-axis represents total experimental duration of open field test.

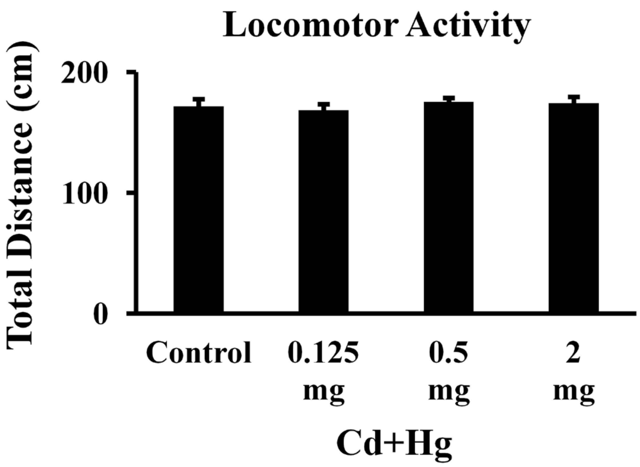

Figure S2: Locomotor activity of male offspring at eight weeks of age. Total movement distance in the open field test system was tested ($P>0.05$ compared to controls; $n = 8$ per treatment). Data are presented as means ± SEMs.

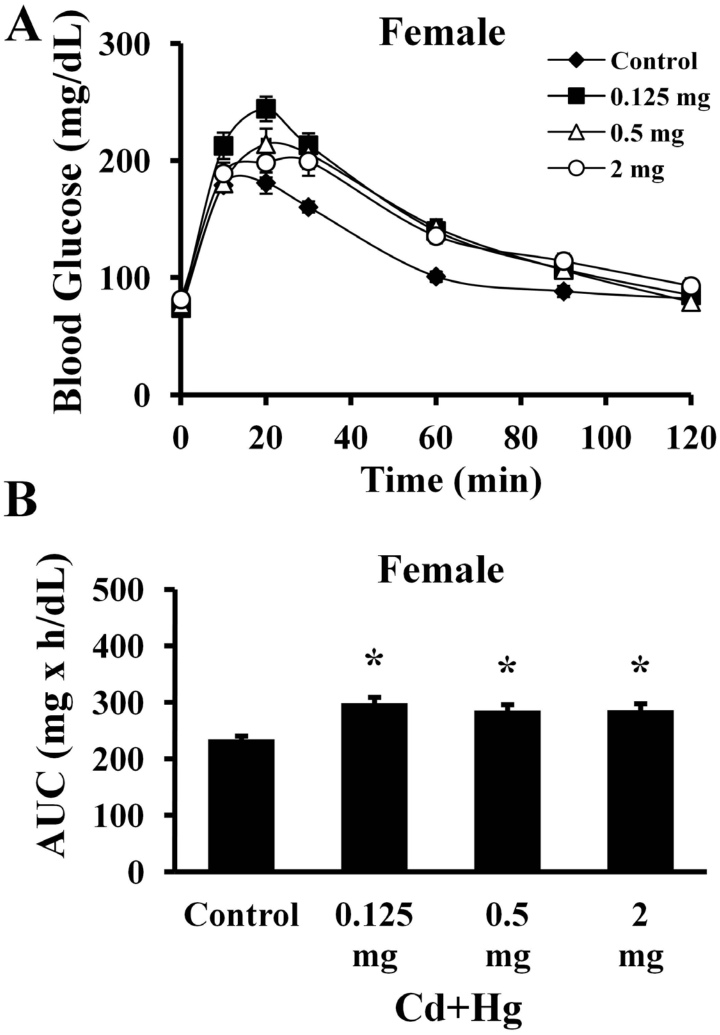

Figure S3: Glucose tolerance test and area under the curve values for 12-week-old female offspring. (A) Glucose tolerance of female offspring (n=14 per treatment). X-axis represents experimental duration in minutes and Y-axis represents blood glucose concentration in mg/dL. Significant differences over time between treatment versus control offspring were tested (P<0.05 compared to controls). (B) Area under the curve values (* P<0.05 compared to controls). X-axis represents each treatment group. Y-axis represents AUC values for plasma glucose in mg x h/dL of blood. Data are presented as mean ± SEM.

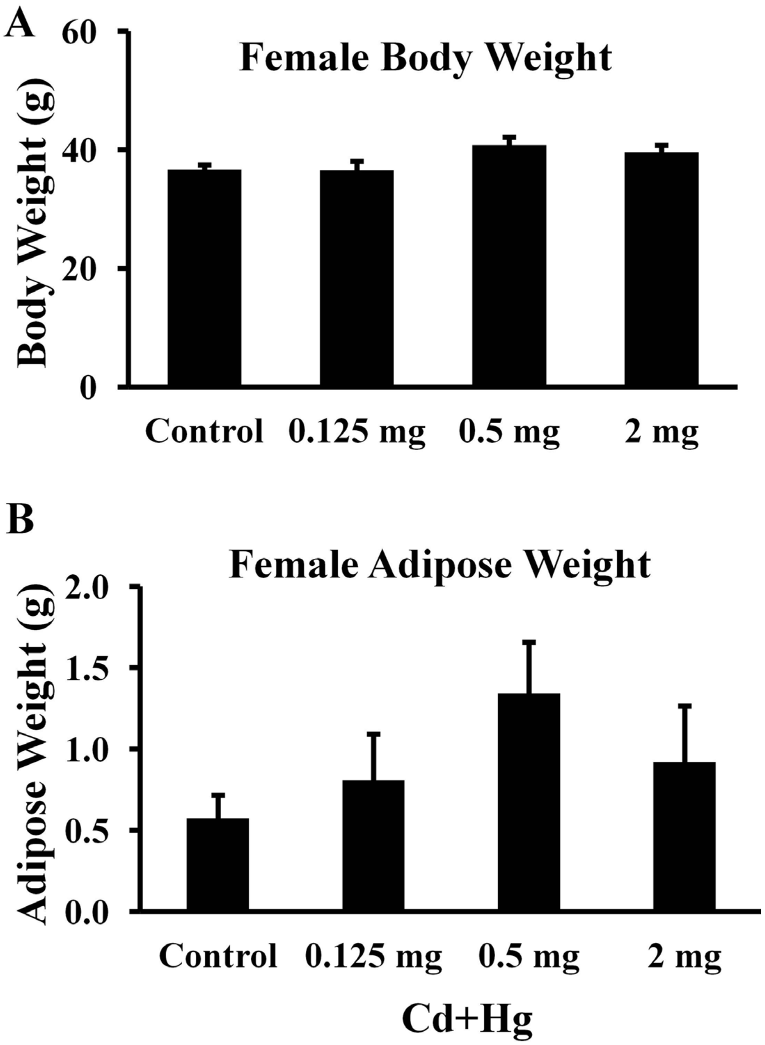

Figure S4: Body and adipose weights of female offspring at 24 weeks of age ($P>0.05$ compared to controls; n=14 per treatment). (A) Female offspring body weights. (B) Female offspring abdominal adipose weights. X-axis represents each treatment group. Data are presented as mean ± SEM.
